# LFA-1 in T Cell Migration and Differentiation

**DOI:** 10.3389/fimmu.2018.00952

**Published:** 2018-05-03

**Authors:** Brandon L. Walling, Minsoo Kim

**Affiliations:** Department of Microbiology and Immunology, David H. Smith Center for Vaccine Biology and Immunology, University of Rochester, Rochester, NY, United States

**Keywords:** T cell migration, integrins, LFA-1, chemokines, T cell activation, T cell differentiation

## Abstract

Maintenance of homeostatic immune surveillance and development of effective adaptive immune responses require precise regulation of spatial and temporal lymphocyte trafficking throughout the body to ensure pathogen clearance and memory generation. Dysregulation of lymphocyte activation and migration can lead to impaired adaptive immunity, recurrent infections, and an array of autoimmune diseases and chronic inflammation. Central to the recruitment of T cells, integrins are cell surface receptors that regulate adhesion, signal transduction, and migration. With 24 integrin pairs having been discovered to date, integrins are defined not only by the composition of the heterodimeric pair but by cell-type specific expression and their ligands. Furthermore, integrins not only facilitate adhesion but also induce intracellular signaling and have recently been uncovered as mechanosensors providing additional complexity to the signaling pathways. Among several leukocyte-specific integrins, lymphocyte function-associated antigen-1 (LFA-1 or α_L_β_2_; CD11a/CD18) is a key T cell integrin, which plays a major role in regulating T cell activation and migration. Adhesion to LFA-1’s ligand, intracellular adhesion receptor 1 (ICAM-1) facilitates firm endothelium adhesion, prolonged contact with antigen-presenting cells, and binding to target cells for killing. While the downstream signaling pathways utilized by LFA-1 are vastly conserved they allow for highly disparate responses. Here, we summarize the roles of LFA-1 and ongoing studies to better understand its functions and regulation.

## Introduction

Precise spatial and temporal regulation of adhesion and de-adhesion is critical for immune cell development, localization, and pathogen clearance. LFA-1 is a key T cell integrin that plays a critical role in the regulation of these functions. With this highly diverse set of roles, it is unsurprising that LFA-1 has been implicated in numerous autoimmune and inflammatory conditions including inflammatory bowel disease, psoriasis, diabetes, and arthritis (1, 2). Intriguingly, intracellular signals dictating LFA-1 activation are highly conserved between migration, T cell activation, and cytolytic activity suggesting that any alterations in the signaling may cause substantial biological consequences during the host immune responses. This review will discuss our current understanding of the role of LFA-1 during T cell activation, effector functions, and memory formation.

## LFA-1 Structure

**Figure 1:**
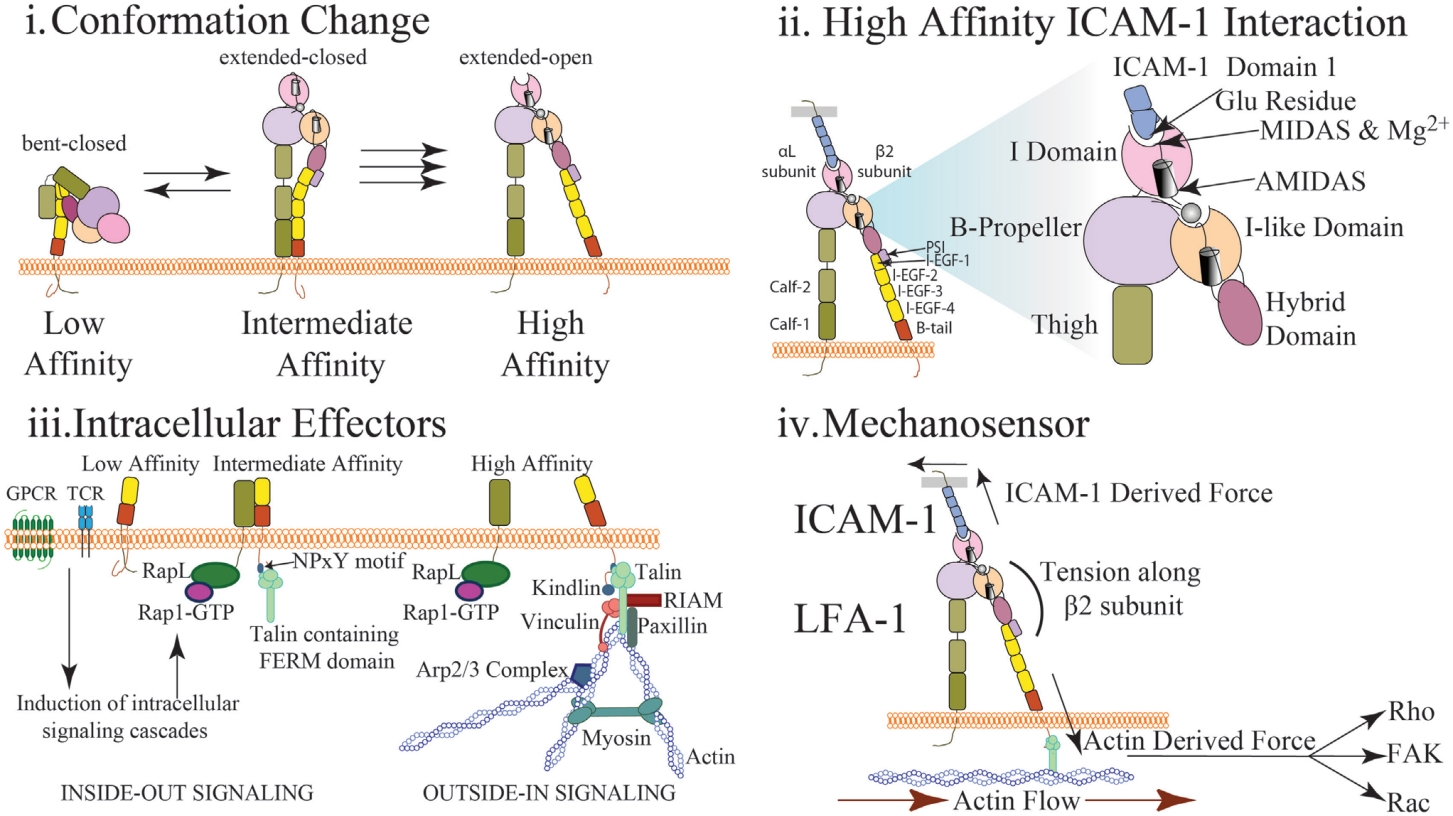
Multidimensional regulation of LFA-1 affinity (i) LFA-1 affinity regulation is mediated *via* conformational changes to LFA-1 structure. In the low affinity state, the bent conformation causes the ligand binding αI domain to be inaccessible to interact with ICAM-1. In the intermediate affinity state, the extracellular leg domains are straightened allowing for low affinity interactions between LFA-1 and ICAM-1. Importantly, the intracellular domains of LFA-1 are not separated and the metal ion-dependent adhesion site (MIDAS) binding site closed. In the high affinity state, disruption of the salt bridge between the α and β cytosolic tails results in conformational shift along the β subunit and αI domain resulting in high affinity LFA-1 *via* the opening of the ligand-binding site. (ii) The αI domain contains the MIDAS within which resides Mg^2+^ coordinating the binding pocket. This site interacts with the glutamic acid-34 in Domain 1 of ICAM-1 to facilitate binding. This induces a shift in the α7 helix to cause the hybrid domain to swing out further stabilizing LFA-1 structure. Additional sites surrounding the MIDAS such as AMIDAS and ligand-induced metal-binding site assist with coordination of the binding pocket and stabilization of high affinity LFA-1. (iii) Upon T cell receptor or chemokine activation, RAP1-GTP recruits a number of factors including RAPL that interact with the α subunit of LFA-1 to induce integrin activation (inside-out signaling). Similarly, talin cleavage allows the FERM domain to interact with the NPxY motif of the cytosolic tail on the β subunit. This interaction causes a dissociation of the salt bridge inducing cytosolic tail separation. Kindlin also contains a FERM domain and interacts with the β subunit to further stabilize high affinity LFA-1. Molecules such as RIAM, talin, paxillin, and vinculin may interact with the cytosolic tails to recruit additional effector molecules and promote a scaffold to interact with actin and reinforce LFA-1 activity (outside-in signaling). Arp2/3 will promote continued actin filament growth while MyH9 functions to provide stress on actin fibers to induce LFA-1 dissociation from ligand. (iv) Interaction of LFA-1 with ICAM-1 and β-actin allows for force driven responses along the β subunit. Transmission of force (arrows) along the β-subunit has been measured in pN scale with actin flow functioning to direct the orientation and location of LFA-1 both at the immunological synapse and during cell migration. Stabilization of the integrin in the high affinity conformation *via* force generation requires adhesion to both the cytoskeleton and ICAM-1. The stiffness of the substrate may also alter the level of force generated thus altering the signaling response. Downstream signal is induced *via* outside in signaling generated through the stabilization of high affinity LFA-1. Phosphorylation of focal adhesion kinase through force generation may play a role in mediating cell adhesion and proliferation. Rho signaling, and thus actin polymerization, may also be altered through changes in force generation resulting in changes in actin dynamics and cell migration. Induction of Rac and CDC42 may also be altered through force generation resulting in changes to cell proliferation and survival.

LFA-1 is composed of α- and β- subunits that together form a heterodimer expressed at the cell surface. These subunits include long extracellular domains, a single transmembrane domain, and short cytoplasmic tails (Figure 1). The extension of LFA-1, which resembles a switchblade-like motion, requires substantial changes to the conformation of both subunits (3). LFA-1 has at least three separate conformational states that are conferred by movement of the extracellular and cytosolic domains: (1) closed/bent, where the integrin has low affinity for ligand and is conformationally unavailable to bind ligand; (2) closed/extended, where the integrin is extended allowing for interaction with ligand, but the cytosolic tails remain closed; and (3) open/extended, where the integrin has high affinity for its ligand and the cytosolic tails have separated (Figure 1i) (3–5).

Roughly half of all integrins, including LFA-1, express an αI domain, which is critical for ligand binding and contains a metal ion-dependent adhesion site (MIDAS) that binds Mg^2+^ to coordinate the binding pocket (Figure 1ii) (3). ICAM-1 will directly bind with the LFA-1 MIDAS and Mg^2+^ by interacting with a glutamic acid residue found in Domain 1 of ICAM-1 (Figure 1ii) (6). LFA-1 is also capable of binding ICAM-2 and ICAM-3 albeit with much lower affinity. Two additional sites, ligand-induced metal-binding site (LIMBS) and adjacent to MIDAS (ADMIDAS), have been shown to regulate cytosolic tail separation and reduce cell spreading, respectively (7–9). Two domains on the α subunit leg, calf-1 and calf-2, have a Ca^2+^ binding loop that is critical to the subunit bending. The β subunit consists of the I-like domain, which is homologous to the αI domain and plays a key role in determining specificity. The hybrid domain, which connects the upper and lower portions of the β subunit, is critical for conformation change. The β subunit leg consists of a plexin/semaphorin/integrin domain that is connected to the βI domain and four integrin epidermal growth factor-like (I-EGF) domains, which facilitate β leg bending (Figure 1i–ii) (3, 5, 8–10).

To facilitate conformation changes, a number of structural modifications occur utilizing the abovementioned integrin components. As the α7-helix is displaced downward during integrin activation, the hybrid domain swings outward leading to separation of the extracellular legs (6). However, due to the flexibility of the extracellular β subunit leg, this separation facilitates only extension of the leg and does not separate the cytosolic tails. Complete cytosolic tail separation requires intracellular effectors, such as talin (Figure 1iii) and stabilization through interaction with actin and ICAM-1 (Figure 1iv). Fluorescent resonance energy transfer (FRET) studies have demonstrated cytosolic tails closely interact under resting conditions, and upon activation, the tails separate and induce an integrin conformational change to high affinity (11, 12). This process through which intracellular signals induce integrin activation is termed “inside-out” signaling. Similarly, integrin adhesion to ligand induces intracellular responses in a process termed “outside-in” signaling. High affinity integrin conformation can be achieved *via* either inside-out or outside-in signaling.

## LFA-1 and T Cell Migration

Extravasation of immune cells from the vasculature is a highly organized process composed of five steps: (1) weak tethering/rolling, (2) firm adhesion, (3) crawling, (4) paracellular or transcellular migration through the endothelium, and (5) migration through the basement membrane (13, 14). Initial adhesion of cells to the endothelium is mediated by the expression of both selectins and addressins (15–19). Upon tethering, cells begin to decrease velocity and roll along the endothelium forming transient low-affinity interactions. Subsequently, immune cells may firmly adhere to the endothelium or be released back into circulation. Firm adhesion to the endothelium is induced by chemokine stimulations and high-affinity integrin activation *via* inside out signaling (20–23). The integrin conformation change can lead to as much as a 10,000-fold affinity increase of LFA-1 to its ligand ICAM-1 (3, 21). ICAM-1 is expressed at low levels and is highly upregulated upon damage or inflammation (24, 25). After secretion, chemokines bind glycosaminoglycan proteins, such as CD44 and syndecans, expressed on the endothelium (26, 27) to subsequently be presented to immune cells. Chemokine and LFA-1 engagement initiate a series of intracellular cascades that induce T cell polarization inducing subsequent migration (28, 29).

LFA-1-mediated adhesion plays essential roles in both naïve and activated T cells extravasation into the lymph node and tissue, respectively (30). Shulman et al. demonstrated that this process can be mediated *via* intra-endothelial chemokine stores at the immune/endothelial cell synapse and surprisingly, that T cell adhesion to the endothelium appeared independent of chemokine stimulation (31, 32). With single-dye tracking and conformation specific antibodies, Bakker et al. demonstrate that in resting monocytes that roughly 5% of LFA-1 is in nanoclusters that are in a fully active state and bound to the cytoskeleton, suggesting that low levels of LFA-1 activation may occur independently of chemokine stimuli (33). It is tempting to speculate from these data that integrin engagement may occur independent of chemokine stimulation suggesting that chemokine may simply act to reinforce integrin engagement and facilitate transmigration. While high affinity interactions are necessary for adhesion, constitutive expression of LFA-1 with the intermediate affinity I-domain led to impaired crawling and diapadesis through limiting detachment at the rear of the cell (34). This demonstrates a need for dynamic regulation of LFA-1 affinity. Indeed, studies have demonstrated that defects in LFA-1 adhesion and activation (changes in conformation, clustering, or cell signaling) through therapeutic treatments and genetic abnormalities can cause deficient immune response and autoimmunity (2, 35–37).

While LFA-1 plays a crucial role in adhesion to the vasculature, very late antigen-4 (VLA-4; α4β1) has also been implicated in T cell extravasation (14, 38–41). In tissues such as the CNS, LFA-1 inhibition is not sufficient to inhibit cell extravasation as cells also utilize VLA-4 (42, 43). However, in other tissues such as the retina, T cell infiltration was LFA-1 dependent and vastly VLA-4 independent suggesting that tissue-specificity plays a critical role in determining integrin-mediated extravasation (44). Indeed, in a bronchial epithelial model, inhibition of LFA-1 lead to a 75% decrease in infiltration whereas inhibition of ICAM-1 or ICAM-2 alone lead to a 50% reduction. However, when both ICAM-1 and ICAM-2 were blocked a 70% reduction in infiltration was observed (45). Additionally, immune cells may alter their integrin dependency. Glatigny et al. demonstrated in T regulatory (Treg) cells that when VLA-4 expression was blocked, cells were still capable of migration utilizing LFA-1 (46). These data demonstrates a highly diverse set of mechanisms, which dictate immune cell infiltration (43).

After firm adhesion, T cells crawl along the endothelium searching for a site to migrate across the endothelial monolayer into the tissue (47). Migration along the endothelium is primarily dictated by chemokine signals that direct cell chemotaxis *via* chemotactic gradients. However, LFA-1-ICAM-1 interaction also plays a critical role in regulating the direction of T cell migration in the blood vessel. T cells and hematopoietic stem/progenitor cells can migrate against shear flow on ICAM-1, while T cells mainly migrate with the flow on VCAM-1 (42, 48, 49). Diapadesis can occur through either paracellular (in between the junction of two cells) or transcellular (through a single endothelial cell) mechanisms. While most cells (~90%) are thought to utilize paracellular migration, the processes dictating para- vs. trans- cellular migration are still being investigated (50, 51). Evidence suggests that ICAM-1 density, monolayer organization (e.g., tricellular junctions), and previous cell diapadesis at the same location (“hot spots”) are all implicated in dictating this phenomenon (50, 52–55). Additionally, cells have been found to survey the tissue with LFA-1/Wiskott–Aldrich Syndrome Protein-dependent protrusions, which have been observed to penetrate as deep as 600 nm into the endothelial cell to promote transcellular migration (56).

Additionally, endothelial cells may facilitate diapadesis *via* the lateral border recycling compartment (LBRC) (54, 57). Mediating changes in endothelial cell junctions to facilitate extravasation, the LBRC has been shown to be essential for transcellular migration. Additionally, numerous diapadesis regulators, including cadherins, CD99, junction adhesion molecules, and platelet endothelial cell adhesion molecules are thought to determine and mediate cell extravasation and are thus a topic of continued research (58–60). We demonstrated that uropod elongation acts as the final step in leukocyte transendothelial migration. During this elongation, CD18^+^ microparticles are left behind which may play a role in either prevention or promotion of leukocyte transmigration at the site (55).

Upon successful migration across the monolayer, T cells utilize a number of β4 and β1 integrins to migrate along the basement membrane composed primarily of collagen and laminin. Intriguingly, the basement membrane appears to be lost directly at the transmigration site (61, 62). While the exact reason for this loss remains under investigation, it is believed to help control cell migration, mediate cell death at the infiltration site, and maintain tissue structure. Following this last step of migration across the endothelial barrier, immune cells continue to migrate through the tissue interstitium to exert their effector function (63).

During T cell migration, LFA-1 engagement is primarily utilized in two-dimensional spaces. One study found that neither LFA-1 nor α_4_ integrins support stable adhesions of naive T cells to neighboring T cells, DCs or stroma in the lymph node T cell zones (64). Indeed, studies have shown that in dense, 3D tissues dendritic cells are capable of migrating without integrin adhesion though actin-polymerization (“flowing”) and myosin II-based contractions (“squeezing”) (65). However, T cells appear to require integrin-mediated adhesion in the tissue microenvironments under inflammation (66). Therefore, it is likely that integrin-mediated T cell migration is determined by integrin/ligand expression and tissue density in which the cell is found. It is also important to note that, while LFA-1-independent migration occurs under depleting conditions within the lymph node, the outcome of the immune response may be altered. Additional studies demonstrated that LFA-1 blockade abolished directed, high velocity migration of naïve T cells (67), suggesting that LFA-1-mediated migration is important for the speed, and the pattern of T cell migration in the lymph node.

In addition to the conformational changes in LFA-1 (see Chapter 1), precise and dynamic regulation of LFA-1 recycling is a key to ensure efficient T cell migration and adapt to the constantly changing microenvironment (68, 69). While LFA-1 recycling occurs constitutively, as much as 75% of all integrins are internalized and redistributed within 15 min of cell migration onset (69). While integrins can utilize both clathrin-dependent and -independent pathways (69), LFA-1-dependent endocytosis is primarily mediated through clathrin-independent, cholesterol-sensitive mechanisms (68) and play an important role both in mediating cell migration and cell polarization through the partitioning of molecules near LFA-1 (68–70). Upon internalization, a series of steps determine the fate of intracellular LFA-1 (degradation vs. recycling). Integrins are generally thought to return to the cell surface through *via* either a direct exocytosis route (*via* Rab4 or Rab5) or the perinuclear recycling compartment route (*via* Rab11) (69, 71, 72). LFA-1 containing vesicles during T-antigen-presenting cell (APC) interactions have also been found to require Arf6 + Rab22 (72). LFA-1 can specifically utilize a Rab13-dependent pathway through which Rab13 associates with Mst1 to facilitate increased integrin activation, as evidenced by increased LFA-1 clustering and cell migration (69, 73). Additionally, LFA-1 recycling requires Rap2-expressing vesicles which work synergistically with Rab13 to mediate new adhesion, while Rap2 facilitates continuous adhesion (74). T cell activation may also be impeded through defects in LFA-1 recycling as demonstrated with Rab13 or Rab27 inhibition (73, 75, 76). However, determining the precise roles of each recycling mechanism during LFA-1-mediated cell migration and activation requires more investigation.

## LFA-1 and T Cell Activation

T cell activation is a highly organized process that can be divided into distinct events described by both T cell motility in the lymph node and T-APC interactions. The first phase is highly dynamic, as immune cells migrate along fibroblastic reticular cells (FRCs) while scanning for antigens. The second phase is defined by low motility and high interaction between the T cells and APCs. The final stage is characterized by regaining a high level of motility, effector differentiation, and proliferation (77).

T cells migrate along FRCs to sample antigens *via* random interactions with antigen-bearing dendritic cells. These short, transient interactions, termed kinapses, are characterized by reduced T cell migration (78). Interaction with APC is determined *via* affinity between the peptide-MHC (pMHC) complex and the T cell receptor (TCR). As these interactions are low affinity (1–100 nM Kd), they are highly specific for only 1 × 10^5^–1 × 10^6^ TCRs (79, 80). Upon recognition of cognate antigen, a T cell ceases migration and induces surface and cytosolic changes in both the APC and the T cell. This phenomenon is defined as the second phase of T cell activation and is characterized by a loss of motility, extended T cell/APC interaction and the formation of an immunological synapse (IS) (77, 78). These changes include a loss of polarity in the T cell and surface molecule reorganization referred to as supramolecular activation clusters (SMACs). The IS can be segregated into three distinct portions similar to a bullseye pattern. The center of the bullseye, aptly named center-SMAC, contains the TCR/CD3 complex and the co-stimulatory molecules CD28 and PKCθ. The outermost ring, or the distal ring (d-SMAC), is composed of the phosphatase CD45, and the center ring, or peripheral SMAC (p-SMAC) is composed of LFA-1 and talin (81). Surprisingly, it has recently been observed that under basal conditions, LFA-1 can be found in clusters prebound to the cytoskeleton suggesting this may help to induce initial cell adhesion and formation of nanoclusters upon TCR engagement (33, 82). Interactions at the IS can be defined by the duration of the interaction. Synapse formation (>5 min) and kinapse formation (<5 min) are determined by the affinity of the TCR/pMHC complex and the activation phase of the T cell. Evidence suggests that both types of interaction are required for complete T cell activation to balance activation signals imparted from the APC with differentiation and proliferation. Additionally, dysregulation of this process may lead to tolerance or autoimmunity through altering the balance of activation signals the T cell receives or through altering the affinity threshold required to engage TCR/MHC complexes (78).

Interaction between TCR and pMHC induces a phosphorylation-mediated signaling cascade. This process activates LFA-1 at the IS leading to firm adhesion required for effective T cell activation. LFA-1 may be activated through a number of pathways that convert the small GTPase RAP1 to its active GTP bound form (83–85). Importantly, RAP1 activation is dependent on the context of activation (TCR vs. chemokine) with data demonstrating that RhoH functions as a rheostat with differential localization within the cell leading to alterations in LFA-1 activation (86). Many of these pathways require the LAT signalosome, including the PLCγ1 activation of DAG regulated-guanine exchange factor (GEF)1 (CalDAG-GEF), which directly acts on RAP1. Additionally the adaptor protein CRKII can interact with C3G, a GEF, to activate RAP1. CRKII-C3G can also be activated *via* the WASP family member 2 (WAVE2) actin related protein 2/3 complex (ARP2/3)-ABL complex. Upon conversion of RAP1 from the inactive GDP-bound form to the active GTP-bound form, it interacts with ADAP and the adaptor SKAP55 to recruit RAP1 to the plasma membrane (87). This allows for recruitment of the RAP1 effectors RAPL, Mst1, PDK, and RIAM to induce integrin activation. The RAP1/Mst1/Kindlin3 complex can be formed through inside-out integrin activation signals, but may also play a role in stabilizing outside in signaling (88–91). This process is essential for LFA-1 recycling, as RAP1 complexes play key roles in delivering LFA-1 vesicles to the cell surface (78, 81, 92, 93).

Antigen-presenting cell expression of ICAM-1 is also required for effective T cell activation. ICAM-1 expression on DC’s plays a crucial role in mediating T cell migration and localization throughout the lymph node (94–96). Additionally, ICAM-1 clustering on APC is essential for effective LFA-1 engagement and T cell activation (97, 98). Interestingly, LFA-1 has also been directly implicated by CD8^+^ DCs to facilitate T cell activation *via* acting as a scavenger receptor to collect antigen from antigen-bearing DCs (99).

Disregulation of LFA-1 expression can lead to changes in T cell activation and differentiation (28, 100–102). Stable engagement of LFA-1/ICAM-1 is required to re-enforce many pathways for complete T cell differentiation. LFA-1 crosstalk with Notch signaling has been shown to induce IFNγ production and re-enforce Th1 cell functions suggesting that LFA-1 engagement with tissue resident APCs will further strengthen T cell differentiation (103). Similarly, Tregs have been shown to require talin and LFA-1 activation to induce IL-2Rα upregulation, which is required for Treg function (104, 105). Additionally, we recently demonstrated that an intracellular pool of LFA-1 is relocalized to the cell surface upon initial T/APC interactions and plays a key role in T cell memory development (76).

## LFA-1 and T Cell Cytotoxic Response

Fully differentiated effector CD8^+^ T cells kill infected/transformed target cells *via* caspase-dependent apoptosis (106, 107). Upon recognition of a target antigen *via* TCR/pMHC complex formation, CD8^+^ T cell activates LFA-1 and binds to ICAM-1 expressed on the target cell. Important functions of LFA-1 during the cytotoxic response was demonstrated with LFA-1 blockers that inhibited target cell killing (107–109). Cytotoxic T cells form short, LFA-1 driven, kinapse-like interactions with infected cells to facilitate killing with interactions for as little as 10 min inducing apoptosis in target cells (110–112). Similar to the IS formed during T cell activation, TCR-derived (113–115), inside-out signals induce translocation of the microtubule organizing center (MTOC) toward the contact between IS and the target cell. The MTOC and microtubules interact with LFA-1 within the p-SMAC to define the ring shape structure observed during perforin/granzyme release (116–118). Organization of LFA-1 at the p-SMAC is thought to act as a “gasket” to prevent cytolytic granules from escaping (119). Furthermore, studies have indicated that the stability and strength of the LFA-1-mediated contact is critical for effective cytolytic activity (118). Additionally, CTLA-4 signaling has been shown to lead to RAP1-mediated increase in LFA-1 binding (120, 121). While the purpose is unclear, it is possible that this mediates low affinity TCR interactions or in cases of high stimulation, induces greater cell polarization and migration. Finally, galectin coating of the tumor infiltrating leukocytes (TILs) surfaces has led to decreased LFA-1 recruitment and activation at the IS and reduced cytokine secretion, further supporting a key role for LFA-1 in mediating TIL cytotoxic function (109).

## LFA-1 and T Cell Memory Development

As described above, LFA-1 plays a critical role in facilitating naïve T cell activation and differentiation through T cell-APC contacts. Indeed, defects in LFA-1/ICAM-1 interactions have been shown to lead to impairment of memory formation (122, 123). Interestingly, ICAM-1 expression on T cells is important for T cell clustering during transient T–T interactions that provide additional cues for proliferation (124). ICAM-1 deficient cells resulted in higher levels of IFN-γ and granzyme B as well as, increased KLRG-1 expression suggesting increased differentiation toward short-lived effector cells (97). LFA-1 expression is required for the retention of tissue resident memory cells in the hepatic sinusoids and facilitate their migratory patterns unlike skin resident memory cells that are largely sessile (125). Additionally, numerous allograft rejection model studies have demonstrated both in mice and non-human primates that LFA-1 blockades reduce or delay memory cell mediated rejection (126–129). While the precise mechanism of this appears to be a combination of infiltration, proliferation, and cytokine secretion, this demonstrates LFA-1’s multifaceted role in memory T cell function. Finally, it is important to note that this is not exclusive to LFA-1 as studies have suggested the integrin VLA-1 is required for memory T cell development in the airway against influenza infection (130, 131).

Intriguingly, several studies have shown that LFA-1, along with CD8, CD3, and CD43, are asymmetrically inherited into the two daughter (proximal vs. distal) cells upon initial T cell division (132, 133). This has been further studied with fate-associated factors such as IL-2Rα, IFNγR, and T-bet all being asymmetrically distributed (133). Importantly, we demonstrated that unequal inheritance of LFA-1 in daughter cells caused differences in migration, T-APC contacts, tissue retention, and effector functions (76). This study further demonstrated that the unequal inheritance of LFA-1 plays an important role in memory generation and differentiation of T cells into both effector and memory subsets.

## LFA-1 as a Mechanosensor

Recent evidence suggests a role for LFA-1 as a mechanosensor affecting cell signaling and integrin activation through the force generated by ligand binding. For example, it has been proposed that integrin adhesion occurs through a “catch bond” in which as the tension at the ligand binding site increases, the affinity also increases (134, 135). Recent work with a FRET-based LFA-1 tension sensor demonstrated significant tension across the β subunit of LFA-1 upon ICAM-1 binding resulting in the stabilization of active LFA-1 (136). Importantly, force generation requires adhesion to both ICAM-1 and actin to result in increased integrin constraint, cell tension, and cell signaling (33, 98, 136, 137). Actin remodeling *via* WASP-dependent mechanisms is essential for the assembly and distribution of high affinity LFA-1 clusters at the IS. The control of LFA-1 topology at the IS by WASP is related to both the control of the CD4^+^ T cell stop signal (138) and the CD8^+^ T cell cytotoxic activity (139). Further work has demonstrated that retrograde actin flow dictates LFA-1 orientation when bound to ICAM-1 on migrating Jurkat T (137). Similarly, TAGLN2 dependent inhibition of actin depolymerization is required for stable IS (140). Unsurprisingly, this force generation has been shown to be an important part of IS formation, cell cytotoxicity, and may modulate cell migration (42, 136, 141–143). As described in reviews by Sun et al. and Gauthier et al., the role of integrin tensile force requires continued study to fully elucidate its functions on in T cell activation, migration, and cytotoxicity (144, 145).

## Conclusion

As this review has shown, LFA-1 functions are extremely varied but play a critical role in facilitating effective immune responses. Our understanding of the mechanisms through which LFA-1 mediates immune cell function have grown exponentially, yet many questions still remain. As our understanding grows, our capability to modulate this highly adaptive molecule to better treat autoimmunity, cancer, and allograft rejection will continue to improve.

## Author Contributions

BW and MK both researched the topic, wrote and edited the manuscript, and made the figure for this manuscript.

## Conflict of Interest Statement

The authors declare that the research was conducted in the absence of any commercial or financial relationships that could be construed as a potential conflict of interest. The handling Editor declared a shared affiliation, though no other collaboration, with the authors.
